# Supplementary material for: Causal Relationship between Aging and Anorexia Nervosa: A White-Matter-Microstructure-Mediated Mendelian Randomization Analysis
Source: Biomedicines. 2024 Aug 16;12(8):1874. doi: 10.3390/biomedicines12081874 (PMC11351342; doi:10.3390/biomedicines12081874)
Supplement: Supplementary file 1 [file biomedicines-12-01874-s001.zip › Supplementary Figure/Figure S8/Leaveoneout plot of MR analysis for TL to ubm-b-2126.pdf]

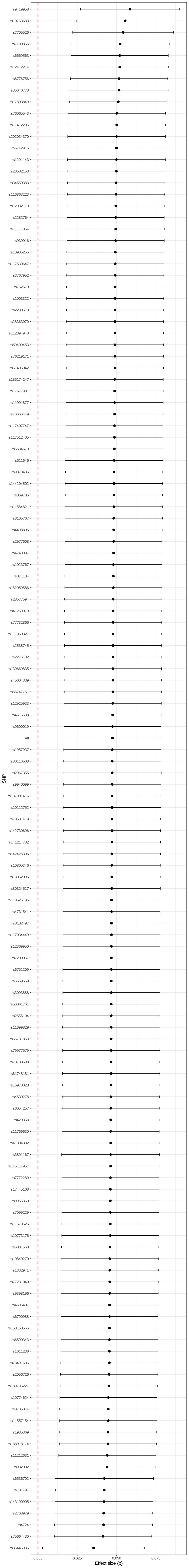
rs9419958
rs10768683
rs7705526
rs7790856
rs6669563
rs12412214
rs6776756
rs35640778
rs17803849
rs76065543
rs11412296
rs202034370
rs5742915
rs1291143
rs28502153
rs34550383
rs116863223
rs12932178
rs2282764
rs11117354
rs939916
rs10905255
rs117630647
rs3767952
rs762679
rs1003322
rs2293579
rs28363070
rs112394943
rs59409453
rs76219171
rs61405042
rs185174247
rs17677991
rs11991877
rs76666449
rs117407747
rs117512405
rs6584579
rs611646
rs9878436
rs144204502
rs869785
rs11584821
rs8105767
rs4498805
rs2977608
rs4743037
rs1023767
rs871134
rs182059586
rs28577594
rs41269079
rs77732866
rs111950327
rs2538745
rs2276182
rs139669835
rs45604339
rs55747751
rs12925933
rs4616688
rs9600019
All
rs1957937
rs80116508
rs2967355
rs9940099
rs137901416
rs10112752
rs73581419
rs142730696
rs141214782
rs142426306
rs10805346
rs13062095
rs80324517
rs113525195
rs4731541
rs8102497
rs117034449
rs12369950
rs7209057
rs6751209
rs6659669
rs3093888
rs56061761
rs2555104
rs11699829
rs66731853
rs79977579
rs73730598
rs61748181
rs16978028
rs4530278
rs6054257
rs429358
rs11769630
rs41304832
rs3891167
rs145114957
rs7772289
rs17445108
rs9955360
rs7099229
rs11579626
rs10773176
rs6881568
rs10840270
rs1332941
rs77231040
rs9398196
rs4695407
rs6790988
rs150150565
rs6590343
rs1611236
rs78491606
rs2056726
rs139795227
rs10774624
rs3785074
rs11557154
rs1985369
rs188918174
rs11212631
rs932002
rs6536702
rs131797
rs143190905
rs2763979
rs4724
rs75664430
rs35446936
SNP
0.000
0.025
0.050
0.075
Effect size (b)
